# Piezoresponse force microscopy and nanoferroic phenomena

**DOI:** 10.1038/s41467-019-09650-8

**Published:** 2019-04-10

**Authors:** Alexei Gruverman, Marin Alexe, Dennis Meier

**Affiliations:** 1 Department of Physics and Astronomy, University of Nebraska, Lincoln, NE 68588 USA; 2 Department of Physics, University of Warwick, Coventry, CV4 7AL UK; 3 Department of Materials Science and Engineering, Norwegian University of Science and Technology (NTNU), N-7034 Trondheim, Norway

## Abstract

Since its inception more than 25 years ago, Piezoresponse Force Microscopy (PFM) has become one of the mainstream techniques in the field of nanoferroic materials. This review describes the evolution of PFM from an imaging technique to a set of advanced methods, which have played a critical role in launching new areas of ferroic research, such as multiferroic devices and domain wall nanoelectronics. The paper reviews the impact of advanced PFM modes concerning the discovery and scientific understanding of novel nanoferroic phenomena and discusses challenges associated with the correct interpretation of PFM data. In conclusion, it offers an outlook for future trends and developments in PFM.

## Introduction

The invention of the atomic force microscope (AFM) in 1986 marked a dramatic shift in scientific research by providing a multifunctional toolbox to explore and manipulate functional properties of a wide range of materials at the nanometer scale. For ferroelectrics and other polar materials, the introduction of one of the voltage-modulated versions of AFM—piezoresponse force microscopy (PFM)—has produced a wealth of new opportunities. PFM enables non-destructive visualization and control of FE nanodomains, as well as direct measurements of the local physical characteristics of ferroelectrics, such as nucleation bias, piezoelectric coefficients, disorder potential, energy dissipation, and domain wall (DW) dynamics (see Box 1). With this, PFM has essentially driven the whole field into the realm of the nanoscale ^1^.

Since the publication of the first review book ^2^, the experimental and physical principles of PFM operation have become common knowledge. A number of papers and books provided a comprehensive description of its technical details and gave abundant examples of PFM imaging and modification capabilities ^3–5^. As the field matured, new advanced PFM modes were developed (Box 2). However, the wide application of PFM revealed a growing number of challenges and concerns related to the imaging mechanism, data interpretation, and quantification. While nanoscale domain imaging has been crucial for the initial advent of nanoferroelectric research, it became also clear that a more careful analysis of the PFM image formation mechanism was necessary, along with comprehensive information on the structure, physics, and chemistry of the materials under investigation, to distinguish real effects from artifacts.

This article, instead of describing the experimental issues of PFM and a variety of accumulated data, focuses on new science and discoveries enabled by PFM. After presenting a brief historical overview of the evolution of conventional PFM into a set of advanced modes, it describes the role of PFM in exploration of new emergent phenomena, including DW conductivity, magnetoelectric switching, voltage-free flexoelectric domain control, tunneling electroresistance, domain vertices, and polar vortices. Specific attention is paid to challenges in PFM application related to a variety of electromechanical coupling phenomena and the complex image formation mechanisms. Based on the recent advances and challenges in the field of nanoferroics and other functional materials, this review offers an outlook for future developments and trends in PFM.

Box 1. Main functions of PFM In conventional PFM, domain mapping is performed by scanning the sample surface with the probe in the contact regime while monitoring the local piezoelectric strain generated by a small a.c. (probing) electric bias. The role of the probe is two-fold: it is used both as: (i) an actuator, which allows electric field application through the nanoscale contact with the sample, and (ii) a sensor, which measures the electromechanical response of the sample by monitoring the cantilever mechanical motion (vertical displacement, torsion or bending). The response amplitude is a measure of the effective piezoelectric coefficient *d*_*zz*_, which, within certain conditions, can be related to the polarization magnitude, while the polarization direction can be determined from the PFM phase signal ^1,2^. The nanoscale lateral resolution of PFM is afforded by the use of a small integrated conducting tip with the apex curvature radius typically in the range of 20–30 nm. Domain imaging resolution, which also depends on the elastic and dielectric properties of the sample, surface conditions, indentation force, etc, can be in the sub-10-nm range as was demonstrated for a variety of ferroelectrics ^32^.

**Box Fig. 1.**
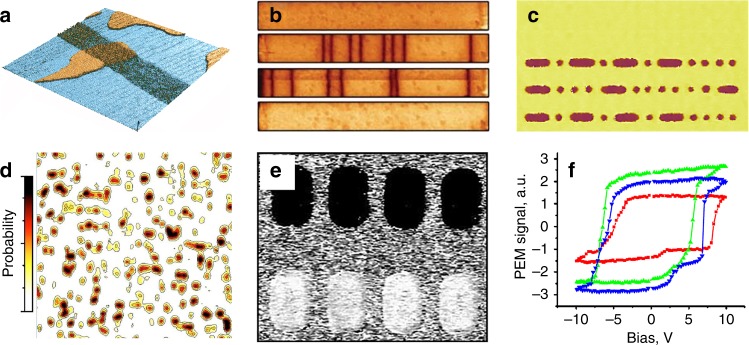
**a** High-resolution imaging of static domain structures: 50-nm-thick PbTiO_3_ film with ac domains (the image size is 5 × 5 µm^2^). **b** Investigation of domain wall dynamics (adapted with permission from ref. ^75^); **c** high-density data storage: electrical writing of stable domains with the characteristic size <40 nm in a BaTiO_3_ thin film (the image size is 1.50 × 0.85 µm^2^); **d** investigation of fast domain switching kinetics: visualization of nanodomain nucleation during polarization reversal in an epitaxial Pb(Zr,Ti)O_3_ ferroelectric capacitor (the image size is 6 × 6 µm^2^) (reprinted from ref. ^93^, with the permission of AIP publishing); **e** Polarization control in µm-scale thin film Pb(Zr,Ti)O_3_ capacitors: upper row capacitors poled by negative voltage pulses, bottom row capacitors poled by positive pulses (the image size is 5 × 6 µm^2^) (reprinted from ref. ^94^, with the permission of AIP publishing). **f** Spectroscopic testing of local switching parameters: hysteresis loops acquired at different locations on the ferroelectric surface (reprinted with permission from ref. ^3^)

**Fig. 1:**
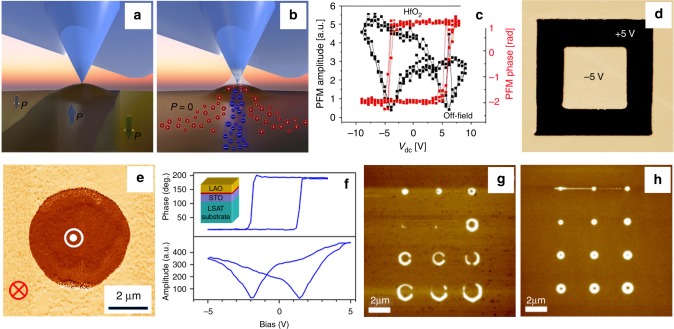
Ferroelectric and non-ferroelectric contributions in PFM. **a** Owing to the linear coupling between piezoelectric activity and spontaneous polarization in the low-field range, ferroelectric domain imaging can be carried out by detecting the electrically induced surface displacement due to converse piezoelectric effect. **b** In addition to the mechanism mentioned in **a**, other mechanisms may contribute to the image formation in PFM in ambient conditions: electrochemical reactions facilitated by the water meniscus at the tip–sample junction, field-induced migration of anions/cations, surface charging, and carrier injection, as well as non-linear high-order strain effects. **c** Switching spectroscopy PFM on amorphous non-ferroelectric HfO_2_ films yields hysteretic behavior in the PFM phase and amplitude signals. (Reprinted with permission from ref. ^42^. Copyright 2015 American Chemical Society). As both ferroelectric and non-ferroelectric materials can exhibit hysteretic behavior in switching spectroscopy PFM, the observation of such loops alone is insufficient to confirm the ferroelectric behavior. **d** Non-ferroelectric signals due to charge injection and electrostatic forces can mimic the ferroelectric behavior in PFM measurements of non-ferroelectric LaAlO_3_ films. This image demonstrates reversible and long-living PFM phase contrast, which arises after applying a ±5 V d.c. bias to the tip. The image size is 8 × 8 µm^2^. (Reprinted from ref. ^35^, with the permission of AIP Publishing). **e** Electrically switchable and stable PFM phase contrast in Pt/LaAlO_3_/SrRuO_3_ capacitors is attributed to electrically induced oxygen vacancy migration. (Reprinted with permission from ref. ^81^). **f** Redistribution and accumulation of oxygen vacancies either on the surface or at the interface can cause hysteretic field-dependent behavior of the PFM signal in LaAlO_3_/SrTiO_3_ heterostructures. (Reprinted with permission from ref. ^36^. Copyright 2012 American Chemical Society). **g**, **h** Effect of environment in PFM manifests itself in creating different boundary conditions for domain writing and retention. PFM images of domains generated by voltage pulses under ambient conditions (**g**) and low humidity (**h**) reveal drastically different domain sizes and shapes (**g**, **h** are both reprinted from ref. ^47^, with the permission of AIP Publishing)

The important role of PFM in the field on nanoferroics is determined by its ability to perform the following main functions: (1) high-resolution imaging, (2) nanoscale property manipulation, and (3) measurements of local parameters (Box Fig. 1)^3,95^. PFM has allowed mapping of nanoscale domain structures in FEs, primarily thin films, detection of the ultimate size limit for FE domains, delineation of unconventional domain configurations, such as closure domains, and their formation with respect to the electrical and mechanical boundary conditions. An electrically biased tip has provided unrivaled possibilities for manipulating polarization states at the nanoscale, DW engineering, and the acquisition of quantitative data related to domain switching dynamics. Monitoring the sample response as a function of the applied bias, frequency, and time facilitated measurements of the local physical characteristics of FEs, such as piezocoefficients, nucleation bias, disorder potential, energy dissipation, and more.

Box 2. Advanced modes of PFM Recent years have witnessed the development of advanced PFM modes, which have turned PFM into a mainstream experimental method for nanoferroelectric studies. Some of these modes are described below.

***Switching spectroscopy PFM (SS-PFM)***

This method enables nanoscale mapping of switching parameters by acquiring the local PFM hysteresis loop at each point in the PFM image ^19^. Real-time analysis of the spectroscopic data provides information on the spatial variations of the local switching parameters such as coercive bias, imprint, and nucleation bias, which are presented as 2D maps and related to the microstructure and compared with the corresponding macroscopic switching characteristics.

***Stroboscopic PFM (S-PFM)***

This mode provides a possibility of ns-time-resolved studies of domain structure dynamics in ferroelectric capacitors ^21, 93^. This method is based on the visualization of instantaneous domain configurations developing in FE capacitors during step-by-step polarization reversal. The domain switching characteristics, such as nucleation rate and DW velocity, can be directly measured from a set of PFM snapshots taken at different time intervals.

***Vector PFM (V-PFM)***

The use of this mode facilitates nanoscale imaging and characterization of the orientation dependence of the EM response with respect to polarization, piezoelectric constants, and crystallographic orientation of the sample. It allows semi-quantitative evaluation of the piezoelectric tensor elements in samples with known crystallographic orientation and 3D reconstruction of polarization through acquisition of three independent PFM signal components—one vertical (out-of-plane) and two orthogonal lateral (in-plane)—provided that PFM vertical and lateral sensitivity are properly calibrated^23^. Later modification, angular-resolved PFM^96^, enables additional functionality such as discrimination between different ferroelectric domains.

***Resonance-enhanced PFM (RE-PFM)***

This mode overcomes the limitations of conventional PFM in testing materials with intrinsically weak piezoelectric properties and ferroelectrics with a low coercive field or breakdown voltage (ultrathin films and superlattices). The sensitivity is enhanced by several orders of magnitude by employing a piezoelectric excitation at frequencies close to resonance^34^. Of particular importance is the dual a.c. resonance tracking (DART) PFM mode^33^, which alleviates the problem of the feedback-loop instabilities associated with spatial variations of polarization, morphology, and elastic properties.

***Metrological PFM (M-PFM)***

This approach allows quantification of the piezoelectric sensitivity by combining an optical beam deflection method with laser Doppler vibrometry to enable accurate measurements of the cantilever velocity and displacement^97^. It directly measures the tip displacement instead of angular changes in the cantilever shape, as in conventional AFM, allowing greater reproducibility and accuracy in testing the local EM properties. However, as discussed in the main text, even this mode does not ensure quantification of the piezoelectric constants.

***Band-excitation PFM (BE-PFM)***

BE-PFM is based on exciting and monitoring the EM response within a continuous frequency band rather than at a single frequency and allows quantitative measurements of energy dissipation through determination of the *Q*-factor of the cantilever-sample system^98^. Importantly, it also provides information on the frequency dependence of the piezoelectric properties and FE switching parameters.

## A brief history of PFM

A key element of AFM that brought about the possibility of high-resolution characterization of materials is the highly localized interaction between the probing tip and the sample^1,2^. In the case of ferroelectrics, a wide range of polarization-dependent properties, such as elastic, structural, electrochemical, pyroelectric, and piezoelectric, makes them a perfect material system for testing various AFM-based methods sensitive to the electrostatic, mechanical, and/or electromechanical (EM) tip–sample interactions^4^. Application of PFM to ferroelectrics makes use of their piezoelectric activity and its linear coupling with polarization in the low-field range. The underlying physical basis of PFM is similar to the idea of piezoelectric polarization-sensing proposed by Kaufman in the late 1950s^6^. Owing to the converse piezoelectric effect, application of an a.c. electric field to a ferroelectric sample results in surface vibration. The polarization state can be determined by measuring the magnitude and phase of this piezoelectric vibration signal. Therefore, the term “piezoresponse” was initially coined for this AFM-based technique^7^ although, as it was shown later, the EM coupling mechanism detected by PFM can go well beyond the piezoelectric effect^8^. The first AFM imaging experiments based on the converse piezoelectric effect were performed by Guethner and Dransfeld in 1992; their paper on local poling of the ferroelectric polymer vinylidene-fluoride trifluoroethylene (PVDF-TrFE) can be considered as the birth of PFM^9^. However, even before this seminal paper, Dransfeld’s group had used scanning near-field acoustic microscopy to detect different piezoactivity of the poled and non-poled regions of PVDF-TrFE films^10^. The lateral resolution of 1 µm, quite impressive for that time, was determined by the size of the probe apex. A year before, the same group used scanning tunneling microscope for nanoscale piezoelectric measurements^11^. However, this approach was too challenging to implement due to the high instability of the feedback signal. Thus, attempts to use the piezoelectric effect for local polarization detection were well under way from the early 1990s, building up on physical principles and technical approaches proposed earlier. However, it took several years for PFM to take off and start its way toward becoming a mainstream imaging and analytical tool for ferroelectrics. The PFM modes integrated into commercial AFM systems started to appear on the market in the late 1990s (Seiko Instruments, Park Scientific Instruments).

Historically, the invention of PFM is closely related to the advances in ferroelectricity, which, since its discovery in the 1920s, has passed through a number of well-marked “phases” when specific materials, techniques, or models were particularly popular^12^. Even before the development of scanning probe microscopy, it was predicted that the 1990s would be the age of “miniaturization,” when the scaling behavior of FEs would be at the forefront of scientific studies. PFM, along with advances in fabrication of functional ferroelectric nanostructures and theoretical modeling, turned this prediction into reality. Nanoscale visualization of the as-grown domain structure in Pb(Zr,Ti)O_3_ (PZT) thin films^13^ and the demonstration of polarization control in individual PZT nanocells^14^ marked the beginning of a widespread use of PFM and launched the era of nanoferroelectric research. Application of PFM to ferroelectric structures with sub-µm dimensions, such as ultrathin epitaxial films^15^, nanoscale capacitors^16^, and nanotubes^17^, opened a possibility for direct studies of the intrinsic and extrinsic mechanisms of their scaling behavior. The fast progress in PFM applications was further boosted by the demonstration of local spectroscopy measurements—PFM hysteresis loops^13,18^, which allowed nanoscale testing of physical parameters, such as nucleation bias, imprint, piezo-coefficients, and disorder potential. This voltage-dependent PFM approach later evolved into switching spectroscopy PFM (Box 2)—an effective tool for two-dimensional (2D) nanoscale mapping of the local switching characteristics^19^. An important step forward in nanoferroelectric research was the visualization of domain structures through the top electrode^20^. This development allowed the direct observation of domain nucleation and DW dynamics at the sub-100-ns time scale in device-grade PZT capacitors using stroboscopic PFM (Box 2) and provided invaluable information on the scaling behavior of ferroelectric random access memory devices (FeRAM)^21^.

Simultaneous detection of electrically induced contraction/expansion and shear deformation of the sample—the approach later termed as vector PFM (Box 2)^22^—allowed delineation of the in-plane and out-of-plane polarization components and three-dimensional (3D) polarization reconstruction^23^. Expanding usage of PFM stimulated serious theoretical efforts to describe  the image formation mechanism as well as tip-induced domain switching, and to understand the meaning of local PFM hysteresis loops ^24^. Detailed analysis of the sample response and cantilever motion revealed limitations of PFM as a quantitative technique, caused by the nonuniform distribution of the tip-generated electroelastic fields, lack of information on the tip geometry, and complex tip–sample contact mechanics, in addition to the non-trivial sample symmetry/orientation-dependence of the PFM signal^25^.

The mid-00s marked a growing use of PFM in combination with other microscopy techniques further expanding the frontiers of the nanoferroic field. High-resolution imaging of both antiferromagnetic and ferroelectric domains in multiferroic BiFeO_3_ by X-ray photoemission electron microscopy (PEEM) and PFM, respectively, led to the first demonstration of electrical control of antiferromagnetic domains in a single-phase multiferroic^26^. In one of the first examples of simultaneous use of PFM and conducting AFM (C-AFM), Yoshida et al. demonstrated polarization-modulated conduction in PZT thin films^27^. This approach has been utilized to its full extent during the experimental observations of the electroresistance effect in ferroelectric tunnel junctions^28,29^ and enhanced conductivity of ferroelectric DWs^30^. A breakthrough discovery of alternating net magnetic moments at ferroelectric DWs in multiferroic hexagonal manganites has been achieved by using PFM in conjunction with magnetic force microscopy (MFM)^31^.

Around the same time, the utility of PFM in testing the nanoscale EM behavior of a broader range of materials, such as piezoelectric semiconductors and biomaterials, was established. Intrinsic piezoelectricity of biopolymers enabled first nanoscale structural imaging of calcified and connective tissues as well as orientational imaging of protein molecules^32^. Similarly, the polar structure of III–V semiconductors allowed delineation of the inversion domains associated with different termination layers^32^. Extension of PFM usage to materials with weak EM properties necessitated enhancement of PFM sensitivity, which was addressed by the development of the resonant-enhanced mode of PFM by Rodriguez et al. in 2007 (Box 2)^33^ preceded by work of Harnagea et al.^34^.

PFM is still evolving: for example, high-speed and multi-dimensional data acquisition components have been recently added to its already wide portfolio of capabilities. Latest advances are related to the development of metrological PFM and band-excitation PFM (Box 2), which allow multi-frequency spectroscopic analysis of the dynamic processes in ferroelectrics.

## Challenges of PFM

The conceptual simplicity of PFM and the ease, with which the PFM mode can be operated on modern AFMs, should not be mistaken for triviality of PFM data analysis and interpretation. AFM systems tend to turn more and more into “black boxes” that readily produce data with large throughput and high accuracy. On the one hand, the “black-box” design can be an issue for specialists because of reduced flexibility and rather limited options for the development of new experiments. On the other hand—and more problematically—new users are no longer required to have a thorough knowledge or understanding of the experimental details to record PFM data. As a consequence, the danger of misinterpretation and scientifically incorrect conclusions increases. In particular, it is important to remember that PFM is not a foolproof verification tool for ferroelectricity: PFM detects the electrically modulated mechanical strain—a property that is necessary but not sufficient for determining the ferroelectric behavior. The reason is that, in addition to the polarization-dependent piezoelectric deformation, there are several other mechanisms that can lead to a ferroelectric-like PFM response. These mechanisms are discussed below along with other challenges associated with successful experimental implementation and correct interpretation of PFM data.

### Image formation

Independent of a specific PFM mode, image formation always follows the same basic principle. The response of the sample is probed by the tip and recorded with the help of lock-in technique, translating the tip-oscillation into a voltage. The output voltage is then used to generate 2D maps by scanning the tip line-by-line across the sample. PFM is thus by no means a direct measure of ferroelectricity or piezoelectric deformation. Understanding whether the detected signals correspond to the intrinsic ferroelectric properties (Fig. 1a) or potential artifacts is a major challenge.

### Origin of the electromechanical response

Understanding the mechanism behind the EM signal is a key issue for correct data interpretation. For example, charge injection and field-induced oxygen vacancy migration can result in a switchable internal electric field that biases the intrinsic electrostriction, producing a switchable PFM response^35,36^. Electrochemical strain due to ionic migration (Fig. 1b)^37^ is also relevant for mixed ionic–electronic conductors, biosystems, and polymers^38^. In principle, any material that exhibits a macroscopic *D*–*E* hysteresis loop can display hysteretic behavior in PFM^39^. Higher-order EM effects, such as flexoelectricity and switchable electrostriction, can also influence PFM image formation.

### Verification of ferroelectricity

To evidence ferroelectricity, two effects have to be verified: the existence of domains with different orientation of polarization and hysteretic switching between the opposite domain states by electric fields. In PFM studies, it has become standard to present images taken before and after electrical poling to show the existence of switchable polarization domains, while PFM switching spectroscopy is commonly used to measure the corresponding hysteresis loop. However, in PFM, the non-piezoelectric effects mentioned above can convincingly mimic the ferroelectric behavior, producing long-lasting bistable states resembling written domains as well as hysteresis loops even in non-ferroelectric materials^38^. An increasing number of studies on non-ferroelectric materials, for example, TiO_2_^8^ and LaAlO_3_/SrTiO_3_^36^, clearly demonstrate that PFM measurements are insufficient to confirm the ferroelectric behavior (Fig. 1c–f). A striking illustration of PFM limitations in this matter is the ongoing controversy related to the mechanism of the polar behavior in organic perovskites^40,41^. A scanning probe microscopy-based approach has been recently proposed as a possible way to identify PFM artifacts and distinguish between the ferroelectric and non-ferroelectric PFM contributions^42^.

### Calibration

While thorough signal calibration and careful separation of vertical (buckling/deflection) and horizontal (torsion) cantilever excitations allows 3D mapping of polarization, PFM cannot be considered as a quantitative tool for measuring the local piezoelectric coefficients. This limitation is due to a variety of reasons, i.e. non-uniform field distribution, contributions from the non-local electrostatic effects and multiple piezoelectric tensor elements to the EM displacement at a given point; mechanical constraints from the immediate (non-activated) surrounding; unknown contact resistance; etc^43^. In addition, system-inherent background contributions can have a significant impact on the PFM signal^44^. Quantitative PFM evaluation of the piezoconstants can be performed in a limited number of cases, for example, for d_33_ measurements in perovskite (001)-oriented capacitors with a small thickness/area aspect ratio^22^.

### Environmental influence

In addition to the aforementioned intrinsic phenomena, the sample environment and surface effects can be decisive for the outcome of a PFM scan^45^. Under ambient conditions, a water meniscus at the tip–sample contact promotes surface charging and electrochemical reactions, affecting the domain switching and retention behavior (Fig. 1g, h)^46,47^.

### Frictional forces

**Fig. 2:**
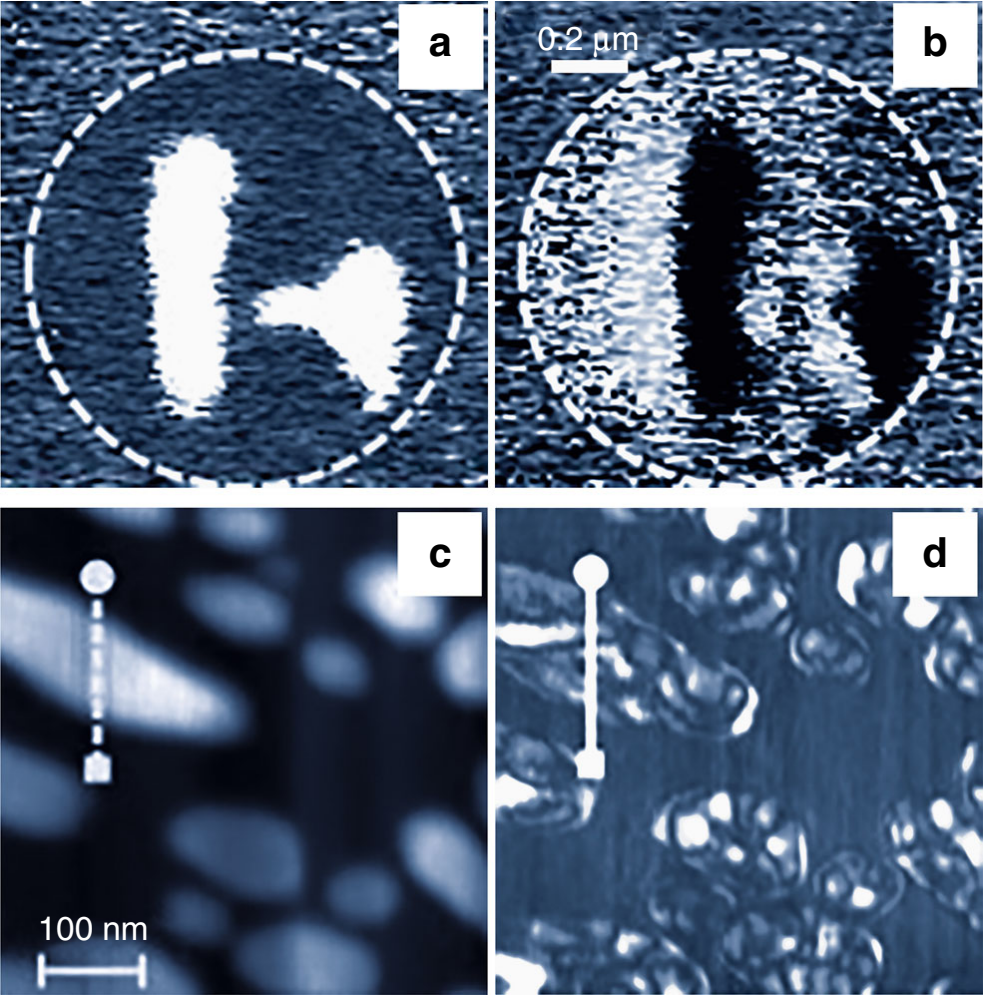
PFM imaging artefacts. **a**, **b** Vertical (**a**) and lateral (**b**) PFM phase images of the 180° domains in a microscale (001)-oriented Pb(Zr,Ti)O_3_ capacitor. Lateral PFM contrast in **b** arises due to the changes of the top electrode morphology induced by the vertical expansion and contraction of the antiparallel 180° domains causing torsional movement of the cantilever, which changes its direction with the change in the surface slope sign. **c**, **d** Any asymmetry in the tip–sample system, for example, due to the surface slope, asymmetric tip apex, local variations in sample stoichiometry, or dielectric or elastic constants, may cause imaging artifacts especially pronounced in lateral PFM. **c** Atomic force microscopic topography and **d** lateral PFM images of the (001)-oriented Pb(Zr,Ti)O_3_ nanograins showing strong variations in the lateral electron microscopic response, which are not related to the in-plane polarization (**c**, **d** are adapted from ref. ^99^, with the permission of AIP Publishing)

Electrically driven shear deformations, which correspond to the off-diagonal elements of the piezoelectric tensor, can be detected by monitoring the lateral PFM signal induced via tip–surface friction forces. However, surface contamination, topographical features, inhomogeneous electrical fields, complex frequency spectra, and mechanical clamping can significantly complicate the interpretation of lateral PFM data and cause imaging artefacts (Fig. 2). As these phenomena collectively affect the friction forces, the lateral PFM signal is a superposition of various contributions rather than a direct fingerprint of the FE domains. This makes lateral PFM essentially unsuitable for studying domains in the ferroelectric capacitors (Fig. 2a, b). Particular caution is required when using lateral PFM to analyze one-dimensional and 2D structures with constrained geometry, including nanoparticles, nanorods, and nanotubes (Fig. 2c, d).

### Standards on PFM measurements

So far, attempts to develop a standardized PFM measuring protocol to gain consistent imaging and spectroscopic results were unsuccessful due to the complexity of the tip–sample interaction and variability of the imaging formation mechanisms as explained above. Although PFM measurements are carried out in a similar way irrespective of the tested materials, PFM practitioners use a broad range of imaging parameters even for the same type of samples: for example, the reported imaging frequencies are in the range from tens to hundreds of kHz. Furthermore, although the conductive cantilevers typically used for FE testing have elastic constants of several N/m, the modulating bias can vary from hundreds of mV up to several Volts. Coordinated efforts by the PFM users through the round-robin comparative spectroscopic testing of the standardized samples, such as periodically poled stoichiometric LiNbO_3_ crystals or (001)-oriented single-crystalline PbTiO_3_ or PZT thin film capacitors, using PFM in conjunction with laser Doppler vibrometry seem to be necessary to identify the data variations attributable to different experimental conditions and setups.

## Discovery of new phenomena and testing polarization-enabled functionality

**Fig. 3:**
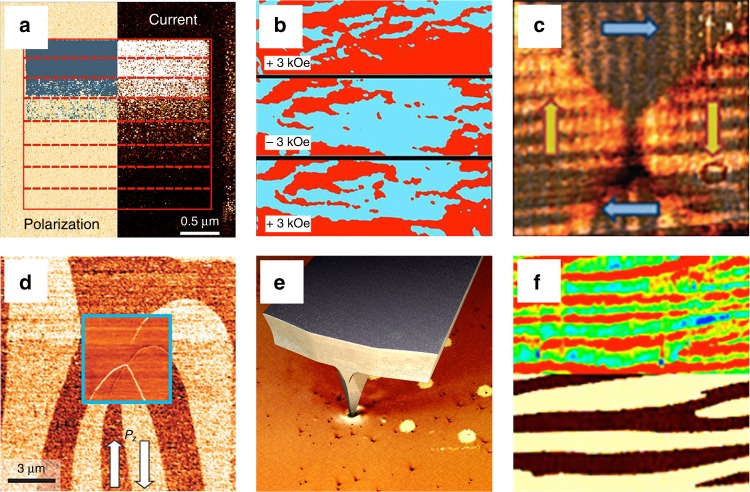
PFM and discovery of novel phenomena. **a** Tunneling electroresistance effect verified by spatially resolved correlation between the onset of polarization reversal revealed by PFM and a resistive switching imaged by C-AFM. A change in PFM contrast correlates with the transition from low current (dark contrast) to high current (bright contrast). (Adapted with permission from ref. ^29^. Copyright 2009 American Chemical Society). **b** Magnetic field control of ferroelectric domains in the multiferroic solid solution of lead zirconium titanate and lead iron tantalite (PZTFT): PFM images reveal a change in the ferroelectric domain structure, which depends on the orientation of the external magnetic field. (Adapted by permission from Springer Nature: ref. ^62^). **c** Emerging topologic domain structures: flux-closure domain formation in a microscale single-crystalline BaTiO_3_ lamella revealed by vector PFM. The image size is 2 × 2 µm^2^. (Reprinted by permission from Springer Nature: ref. ^66^). **d** Domain wall conductivity: the 6 × 6 µm^2^ lateral PFM image obtained on a ErMnO_3_ crystal with arrows indicating the in-plane polarization direction. The inset shows a C-AFM image acquired at the same location revealing different conductance for different types of domain walls. (Reprinted by permission from ref. ^69^). **e** Flexoelectric control of polarization: in ultrathin ferroelectric films, a strain gradient, generated by PFM tip pressure, can produce a flexoelectric field high enough to exceed the coercive field of the film resulting in purely mechanical switching of nanoscale domains. **f** Enhancement of photovoltaic current (C-AFM image in the upper panel) at domain walls (PFM phase image in the lower panel). The size of each panel is 3.0 × 1.5 µm^2^. (Adapted from ref. ^86^ under the terms of the CC-BY 4.0 license)

While at the earlier stages of its use PFM was essential for achieving a nanoscale insight into the ferroelectric switching phenomena, more recently, its combination with complementary nanoscale characterization tools allowed exploration of emerging phenomena in nanoferroic systems. This led to several key discoveries revealing unique electronic, magnetic, and transport properties of nanoferroics (Fig. 3).

### Tunneling electroresistance

One of the most exciting discoveries, involving the use of dual a.c. resonance tracking PFM, was the electroresistance effect in ferroelectric tunnel junctions (FTJs)^29,48^ followed by the demonstration of FE control of the carrier spin polarization in nanoscale capacitors^49^. PFM demonstrated that polar displacements could be stable and switchable in ultrathin films with a thickness down to only several unit cells and established a direct correlation between ferroelectric switching and the electroresistance effect (Fig. 3a). PFM detection of partial polarization switching associated with different tunneling-current levels led to the discovery of the memristive behavior in FTJs^50,51^ opening a possibility for the development of multilevel memories and solid-state ferroelectric-based synapses for neuromorphic computing^52^.

### Multiferroics

Another field that has hugely benefited from PFM is multiferroics. A combination of PFM and complementary methods, such as photoemission electron microscopy (PEEM) and X-ray resonant magnetic scattering, enabled the discovery of a local coupling between FE and antiferromagnetic domains in BiFeO_3_ (BFO) thin films^26^ and the emergence of multiferroicity at the interface between ultrathin BaTiO_3_ films and magnetic layers^53^. In addition, multi-method approaches involving PFM were used to gain control of the exchange bias in BFO films^54^ and to switch local ferromagnetism by electric fields^55^. More recently, PFM and NV-center magnetometry have been combined to gain an insight into the coupling of the ferroelectric and cycloidal antiferromagnetic domains in BFO^56^. In multiferroic bulk systems, combinations of PFM and MFM^31^, magnetoelectric force microscopy^57^, transmission electron microscopy (TEM)^58^, optical microscopy^59^, as well as second harmonic generation^60^, were used to scrutinize the emergence and interplay of structural, electric and magnetic degrees of freedom. In particular, PFM allowed high-resolution imaging of the exotic ferroelectric domain pattern in multiferroic hexagonal manganites^61^. In multiferroic ceramics, PFM together with complementary scanning TEM measurements was used to demonstrate magnetic-field switching of ferroelectric domains at room temperature (Fig. 3b)^62^.

### Topological states

This new, still nascent but rapidly growing field addresses vortices, skyrmions, flux closures, chiral, and other topologically protected states in polar materials. In 2004, unusual vortex states were predicted to occur in nanoscale ferroelectrics due to very high depolarization fields^63^. Just several years later, when advances in processing technologies facilitated the fabrication of such nanometer-scale structures^64^, vector PFM, as a uniquely suited characterization technique, revealed the predicted emergence of vortex domain states and flux closures (Fig. 3c)^65,66^. More recently, nanoscale bubble domains, which can be considered as a precursor of electrical skyrmions, have been resolved^67^.

### Functional DWs

Another emerging field extensively relying on PFM focuses on functional DWs in ferroics^68^, which exhibit a range of unusual physical properties. The work of Seidel et al.^30^ stimulated a worldwide interest in DWs by showing that PFM-written DWs in otherwise insulating BFO thin films were electrically conducting. Anisotropic DW conductance was revealed in the improper ferroelectric ErMnO_3_ (Fig. 3d)^69,70^. Extended studies on other ferroelectrics showed that unusual DW transport was a rather generic property occurring in a wide range of systems. This includes classical (proper) FEs (PZT^71^, BaTiO_3_^72^) as well as improper ferroelectrics RMnO_3_ (R = Y, In, Dy–Lu)^69^ (Ca,Sr)_3_Ti_2_O_7_^73^, Cu_3_B_7_O_13_Cl^74^). The functional properties of DWs and the possibility to inject, erase, or dynamically dope them^75^ offer unique opportunities for building future agile nanoscale devices^76^. In the first proof-of-principle experiments, PFM helped to demonstrate specific DW-based functionality, using them as spatially moveable nanowires^70^, digital switches^77^, and nonvolatile memories^78^.

### Flexoelectricity

Although demonstrated experimentally almost 50 years ago, flexoelectricity—a coupling between a polarization and a strain gradient—started to draw much attention only with the advent of nanotechnology because of larger gradients at the nanoscale^79^. In ultrathin films, a flexoelectric field due to a strain gradient, generated by PFM tip pressure, may even exceed the coercive field leading to purely mechanical switching of polarization (Fig. 3e)^80^. Nanoscale flexoelectricity has been used for tuning 2D electron gas^81^, mechanical control of resistive switching^37^, manipulation of oxygen vacancies^82^, and even for inducing photovoltaic (PV) effects in centro-symmetric materials^83^.

### Photovoltaics

PVs currently experiences renewed interest, triggered by the discovery of abnormal PV effects in ferroelectric thin films^84^. In seminal experiments on BFO, vector PFM was used in combination with macroscopic current–voltage measurements to investigate the impact of the ferroelectric domain geometry on the PV properties^84^. Subsequent in-depth studies^85^ revealed that the emergent above band-gap voltages originate from the bulk PV effect. Furthermore, photocurrents were scrutinized in spatially resolved experiments to clarify the PV/photoconductive properties of the ferroelectric DWs (Fig. 3f)^86^. In the latest development, PFM allowed demonstration of a tip-enhanced PV effect and light-induced ferroelectric switching^87^.

## Outlook and perspective

We are at an inflection point in PFM development and application. On the one hand, PFM has evolved from an imaging technique to a set of advanced methods, which became the prime tools for probing and controlling the static and dynamic properties of nanoscale ferroic structures and devices. On the other hand, a growing range of tested materials revealed PFM limitations: it is now clear that a careful analysis of the PFM image formation mechanism along with comprehensive information on structure, physics, and chemistry of materials obtained by complementary techniques is necessary to distinguish between science and artifacts. Some predictions for future directions and applications of PFM, made several years ago, have not materialized. For example, while PFM can be used as a valuable platform for conducting rigorous and replicable testing of new scientific ideas and device concepts, it is not likely to become part of an evaluation testbed for integrated memory devices or be exploited for bottom–up large-scale fabrication of electronic circuits.

Where will it go from here? Given that the most important breakthroughs in PFM application have been made in combination with other microscopic techniques, it is safe to predict that this trend will continue. It is envisioned that PFM will be extended to multiple stimuli (magnetic, optical, thermal) and combinations of those to induce mechanical strain response at a broad range of temporal scales to discover emerging phenomena and investigate novel functional properties in a variety of material systems. PFM analysis of the electronic processes in ferroics with respect to the field-induced changes in local structure and chemical composition will be greatly facilitated by acquisition of the spatially resolved chemical 2D and 3D maps by nano-Raman microscopy and time-of-flight secondary ion mass spectroscopy—approaches that have just started to gain momentum^88^. One of the main limitations of PFM will remain its relatively low time resolution. Short (~10 µm) cantilevers with high resonant frequencies (up to several MHz) facilitate video-rate PFM with an imaging speed of about 10 frames per second. This will allow investigation of domain structure evolution during thermally stimulated phase transitions and relaxation processes on a 100-ms time scale^89^. However, in application to the fast switching processes, the time resolution is unlikely to go below the current state-of-the-art nanosecond range of stroboscopic PFM being limited by the external circuit time constants. Extension of PFM spectroscopy into the MHz range will enable collection and multivariate analysis of large amounts of data allowing a deeper understanding of the underlying mechanisms of the voltage–time-dependent switching and energy dissipation processes. One of the possible hurdles in big-data analysis could be the streaming rate of the data.

Realization of DW-based nanotechnology requires application of PFM in combination with C-AFM, MFM, scanning impedance microscopy, and nano-Raman spectroscopy complemented by high-resolution electron microscopy studies. This comprehensive approach is desirable to get a better knowledge of the local electronic states, carrier density profile, mobility and conduction mechanisms, and their relation to the local atomic structure. First steps in this direction have already been made and include local Hall effect measurements^90^ and contact-free high-resolution conductance measurements^91^, as well as advanced nonlinear optical experiments^92^. Controlled generation and driving of DWs via electric and strain field gradients is a promising approach for the realization of DW-based devices and undoubtedly will be explored further.

As a technique, PFM presents a new paradigm in exploring and designing emergent electronic properties of advanced oxide, hybrid, and organic nanoferroic materials and nanostructures. State-of-the-art PFM methods, in conjunction with first-principle modeling and atomically controlled growth, will play an increasingly important role in investigating the fundamental issues related to the critical behavior and switching dynamics in ultrathin ferroelectric and multiferroic heterostructures. Furthermore, such methods will allow us to understand the interplay between the ferroelectric and ferromagnetic order parameters, the role of structural defects and interfacial properties in the electronic transport at the nanoscale and the scalability of heterostructures to micro-, meso-, and nano-dimensions.
